# Comparison of Ki67 Proliferation Index in Gastrointestinal Non-Hodgkin Large B-Cell Lymphomas: The Conventional Method of Evaluation or AI Evaluation?

**DOI:** 10.3390/diagnostics13172775

**Published:** 2023-08-27

**Authors:** Miruna Cristian, Mariana Așchie, Mariana Deacu, Mădălina Boșoteanu, Gabriela Izabela Bălțătescu, Andreea Georgiana Stoica, Anca Antonela Nicolau, Ionuț Poinăreanu, Cristian Ionuț Orășanu

**Affiliations:** 1 Faculty of Medicine, “Ovidius” University of Constanta, 900470 Constanta, Romaniacritian.ionut@gmail.com (C.I.O.); 2 Center for Research and Development of the Morphological and Genetic Studies of Malignant Pathology-CEDMOG, “Ovidius” University of Constanta, 900591 Constanta, Romania; 3 Department of Clinical Pathology, “Sf. Apostol Andrei” Emergency County Hospital, 900591 Constanta, Romania; 4 Institute of Doctoral Studies, School of Medicine, ”Ovidius” University, 900573 Constanta, Romania; 5 Academy of Medical Sciences, 030171 Bucharest, Romania; 6 Department of Hematology, ”Sf. Apostol Andrei” Emergency County Hospital, 900591 Constanta, Romania; 7 Department of Pathology, Săcele Municipal Hospital, 505600 Brașov, Romania

**Keywords:** artificial intelligence, computer-assisted image interpretation, pathology, lymphoma, large B cell, diffuse, immunohistochemistry

## Abstract

The most common NHL subtype in SEEU is DLBCL (39%), and it manifests with a variety of cellular morphologies and a high proliferation index. Also, the GI tract is the most common site of extranodal NHLs, and most NHLs involving the GI tract are of B-cell lineage, of which diffuse large B-cell lymphoma is the most common subtype, irrespective of location. The last few years have seen digital pathology as a vital technology that has a positive impact on diagnostics, but studies on the use of DP for lymphoma identification, however, are still restricted to only determining whether a tumor is present or absent. Using the example of cases of malignant NHL, we aim to investigate the diagnostic utility of DP using QuPath software in evaluating the proliferation index and the prognostic significance and to show that improved visualization and analysis contribute to the convergence of these complementary diagnostic modalities for lymphomas. The average proliferation index (Ki67) was 58.33% with values between 10% and 85%. After the stratification of the cases, an increased proliferation index was observed in the majority of cases (53.33%), and this aspect was associated with the advanced age of the patients (*p* = 0.045). Visual assessment provides lower Ki67 values than automated digital image analysis. However, the agreement coefficient between the conventional method and the AI method indicates an excellent level of reliability (ICC1–0.970, ICC2–0.990). The multivariate analysis revealed that in the cases where the proliferation index Ki67 is high (˃70%), the IPI score represents an important risk factor predicting mortality (HR = 10.597, *p* = 0.033).

## 1. Introduction

Diffuse large B-cell lymphoma (DLBCL) is the most common type of non-Hodgkin lymphoma worldwide, representing approximately 30–40% of all cases in different geographic regions [1]. The most common NHL subtype in South-Eastern Europe (SEEU) was DLBCL (39%), followed by FL (15.8%), and the relative frequency of DLBCL is significantly higher in SEEU compared to Western Europe (WEU) (29.3%) and North America (NA) (28.3%) [2]. Diffuse large B-cell lymphoma, not otherwise specified (DLBCL, NOS), is a lymphoma consisting of medium-sized to large B cells with a diffuse growth pattern. This is a morphologically and molecularly heterogeneous entity that does not meet the diagnostic criteria of specific large B-cell lymphoma neoplasms [3]. The most common type of DLBCL, designated as not otherwise specified, represents 80–85% of all cases [1]. High-grade B-cell lymphoma, NOS (HGBL, NOS), represents a heterogeneous category of aggressive mature B-cell lymphomas composed of medium-sized or blastoid cells that do not fit into other defined categories of lymphomas [3]. Most often patients have a rapidly growing tumor mass in single or multiple nodal or extranodal sites [1]. The gastrointestinal (GI) tract is the most common extranodal site involved in non-Hodgkin lymphoma (NHL). At the same time, gastrointestinal NHL cases account for only 1–4% of GI neoplasms. 

With the recent development of digital whole slide imaging (WSI), it is now possible to automatically identify the histopathologic characteristics of lymphomas [4]. The WSI systems help pathologists with microscopic examination by digitizing entire glass slides with stained tissue sections in high resolution [5]. A key factor in achieving the best microscopic interpretation is image quality. Fortunately, the implementation of technology capable of taking data at very fast rates and with outstanding resolution has resulted in a significant improvement in digital picture acquisition in recent years [4]. The Food and Drug Administration (FDA) granted the first WSI system’s commercial permission for digital pathology diagnostics outside the purview of research in 2019 [6]. 

With the development of machine learning techniques, which made significant contributions to the science of pathology and gave rise to a brand-new, extremely complex subject known as “digital pathology”(DP), the image interpretation process of digital slides is actively being explored in diagnostic medicine. One method for defining DP is the process of using whole-slide scanners to convert histology glass slides into high-resolution digital slide images, which are then interpreted and used to generate pathologic data [5].

QuPath is a bioimage analysis software designed to meet the growing need for a user-friendly, extensible, open-source solution for digital pathology and whole slide image analysis [7]. In addition to offering a comprehensive panel of tumor identification and high-throughput biomarker evaluation tools, QuPath provides researchers with powerful batch-processing and scripting functionality and an extensible platform with which to develop and share new algorithms to analyze complex tissue images [7].

Hematopathology has earned a place in the movement toward digitization. Machine learning has recently been used in efforts to diagnose lymphoma with WSI, with encouraging results. Studies on the use of DP for lymphoma identification, however, are still restricted to only determining whether a tumor is present or absent [8,9]. 

The gastrointestinal (GI) tract is the most common site of extranodal non-Hodgkin lymphoma, accounting for 20% to 40% of all extranodal lymphomas. Most non-Hodgkin lymphomas involving the GI tract are of B-cell lineage, of which diffuse large B-cell lymphoma is the most common subtype, irrespective of location [10].

The main purpose of this current study is to investigate the diagnostic utility of DP using QuPath software (version 0.4.3) in evaluating the prognostic significance of Ki67 in patients with gastrointestinal large B-cell lymphomas, to compare the obtained values between the conventional method of evaluation and AI evaluation, and to observe the prognostic role in patient survival of the studied parameters in a group of patients diagnosed within two centers from Romania, observing their associations. Also, we want to show that improved visualization and analysis by using digital pathology contribute to the convergence of these complementary diagnostic modalities for lymphomas. 

## 2. Materials and Methods

We performed a retrospective study over 10 years (2012–2021) that included cases of gastrointestinal non-Hodgkin large B-cell lymphomas. These cases were diagnosed in the hematology department of the County Emergency Clinical Hospital of Constanţa and Sacele County Hospital of Brasov. The inclusion criteria consisted of adult patients and histopathological diagnosis of diffuse large B-cell lymphoma, NOS, and high-grade large B-cell lymphoma, NOS (HGBL), localized in the digestive tract. The exclusion criteria consisted of nodal and other extranodal disease localization except digestive tract (oropharyngeal area, stomach, small and large intestine) diagnosed cases and recurrences. 

The patient clinical observation forms and the hospital’s electronic medical records were analyzed for information about clinical data, paraclinical data, and treatment. 

Following the surgical procedures, the surgical specimens were sent to the pathology department of the same hospital for examination. First, a gross description was made, along with information about the lesions’ greatest diameter, localization, color, and consistency of the specimens. The specimens were prepared and processed according to international protocols within the Clinical Service of Anatomic Pathology, County Emergency Hospital, Constanta, and Service of Anatomic Pathology, Sacele Municipal Hospital, Brasov, and by embedding them in paraffin and applying hematoxylin–eosin on the slides. 

The final diagnosis was made according to the WHO criteria appropriate to the year in which the patients were diagnosed. Two different pathologists reevaluated the relevant cases while taking into account the most recent WHO classification guidelines (5th edition). Following the guidelines outlined in previous studies by Lister et al., all patients were categorized using the Eastern Cooperative Oncology Group (ECOG) Performance Status, Ann Arbor staging system, and International Prognostic Index (IPI) score [11,12].

The immunohistochemical evaluation was performed at the Pathology Department of the County Emergency Clinical Hospital of Constanţa and Sacele County Hospital of Brasov, using CD5, CD10, CD20, MUM1, Bcl6, and Ki-67 biomarkers from Biocare Medical, LLC (Irvine, CA, USA). According to the algorithm developed by Hans et al., tumors were classified as either GCB (germinal center B-cell-like) or non-GCB (non-GCB) subtypes in DLBCL [13]. All patients benefited from the treatment, and the protocol scheme used was CHOP or R-CHOP. The tests were performed on 4-micron-thick paraffin sections according to the manufacturer’s protocols. Before the final signing of the case, a review and reconciliation of the slides took place immediately after each major diagnosis was made on the digital slides.

The scanning of the slides was performed at the Center for Research and Development of the Morphological and Genetic Studies of Malignant Pathology (CEDMOG). The WSIs were obtained using the Huron LE120TM 4000XT scanner (Huron Technologies International Inc., St. Jacobs, ON, Canada). Firstly, the slides were evaluated using a Nikon Eclipse E600 microscope, and secondly, for visualizing the virtual slides, we used Huron ViewerTM Software (Huron Technologies International Inc., Canada) to capture minimum 1000 pixels width/height image patches at random locations on the histologic section, and we used the QuPath Software platform (version 0.4.3) for the quantification of each biomarker. 

The evaluation of the biomarkers using the microscope was carried out qualitatively as well as quantitatively. The quality of the expression was assessed as positive or negative, and the amount of Ki67+ B lymphocytes was evaluated using the average number of Ki67-positive B cells from 10 HPF fields (40×) in the hot spot. According to the data presented in a prior study by Huber F. et al., the objective was to evaluate patients for differences based on immunohistochemically determined Ki-67 values at a cut-off of 70% [14]. Patients with large B-cell lymphomas were retrospectively analyzed in groups of high (>70%) and low (≤70%) Ki-67. 

The quantification of Ki67 using QuPath Software was carried out as follows: batch analysis was applied across all TMA slides to identify the tissue within each core and automatically count the number of positive cells per mm^2^ tissue based upon a fast peak-finding algorithm after stain separation by color deconvolution [7]. QuPath Software, a program for digital image analysis that can reliably and efficiently compute the index by automatically counting cells in the tissue, was used to determine the IHC expression. 

**Figure 1:**
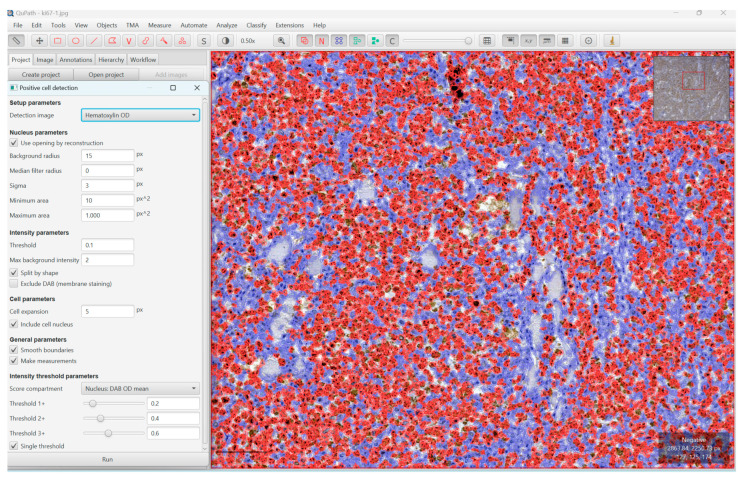
This image shows the counting of positive nuclei stained with Ki67 on the WSI of a DLBCL, NOS, located in the small intestine, using the ”positive cell detection command” of QuPath Software (version 0.4.3).

**Figure 2:**
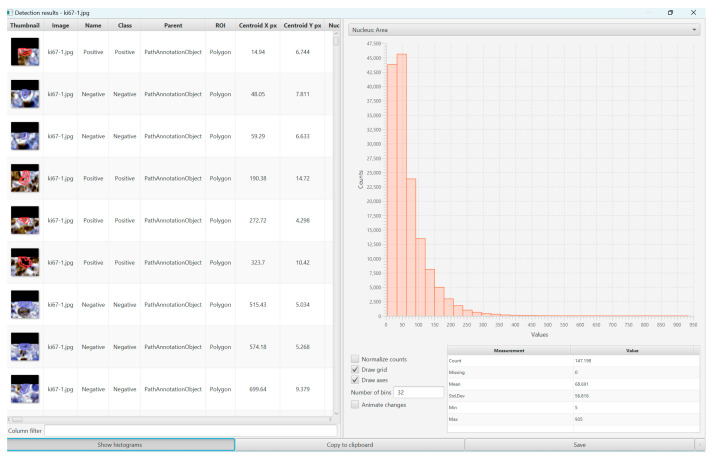
This image shows the detection results (mean value of positive cells) of the WSI of a DLBCL, NOS, located in the small intestine, using the ”positive cell detection command” of QuPath Software (version 0.4.3).

**Figure 3:**
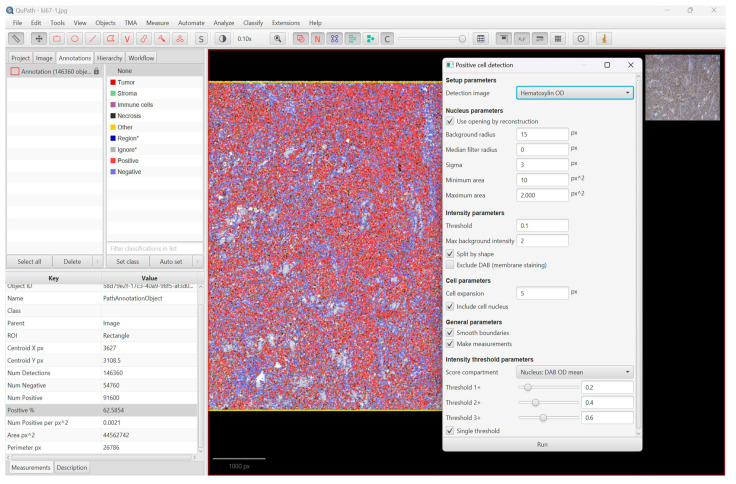
This image shows the detection results (percentage value of positive cells) of the WSI of a DLBCL, NOS, located in the small intestine, using the ”positive cell detection command” of QuPath Software (version 0.4.3).

We ran the command ”cell detection” or ”positive cell detection” after we defined the full area of interest, in our case, the whole Ki67-stained slides, and we used the values provided by the program as the mean value of positive cells and percentage of positive cells (Figure 1, Figure 2 and Figure 3). 

**Figure 4:**
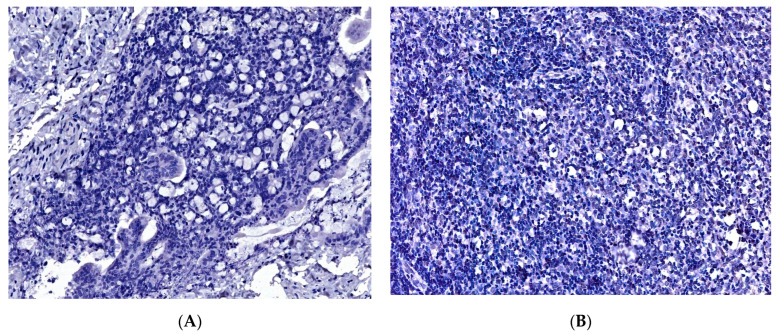

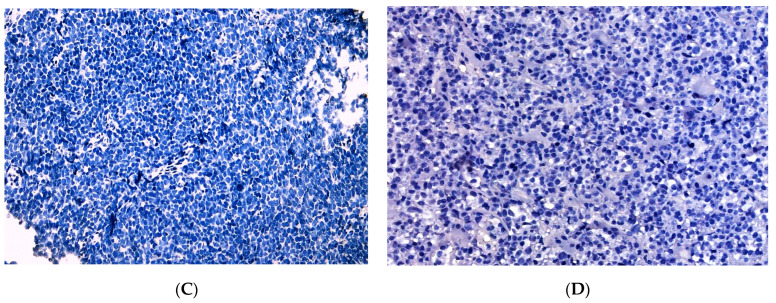
Four representative cases of large B-cell lymphomas with each of the following localizations in the gastrointestinal tract: (**A**) stomach (HE, 40×), (**B**) small intestine (HE, 40×), (**C**) large intestine (HE, 40×), and (**D**) oropharyngeal region (HE, 40×).

Representative WSI sections for these diagnostic categories are shown in Figure 4 (Figure 4A–D). Two sets of 4 representative images were captured for each of the 50 cases, giving a total of 400 images. 

For all tests, statistical significance was set at *p* < 0.05. SPSS software (SPSS version 26.0; Armonk, NY, USA) was used to conduct all statistical analyses. Parameters of interest were the level of LDH at presentation, treatment response, and overall survival (OS). Categorical variables were analyzed using either a chi-squared test or a Fisher’s exact test. Mann Whitney U Test and Kruskal Wallis were used for continuous data as appropriate. To observe the correlations between the studied parameters, we used the Pearson test. For survival analysis, Kaplan–Meier and log-rank tests were used. 

A multivariate linear regression analysis was performed, including gender, age, Ki-67 ≤ 70% or >70%, IPI score, ECOG status, and LDH values. Also, we performed uni- and multivariate analyses to incorporate the clinical prognostic features that make up the IPI score and to confirm the independent effect of cell proliferation on survival because the clinical measures of prognosis reflected by the IPI score can be better predictors of survival than proliferative activity [1]. We calculated the pooled HRs and the 95% confidence interval (CI) to analyze the aggregated impact of Ki-67 expression on the IPI score of lymphoma. HRs and their 95% CIs were calculated using the extracted data with methods according to Parmar’s study [15,16]. 

The inter-rater reliability coefficient was used to evaluate the concordance of the analyses between the conventional method and AI. The analysis was performed on a specific sample (two-way mixed effects) analyzed by two pathologists and the QuPath program (k = 3) following the absolute agreement coefficient.

Our study was approved by the Ethics Committee of both hospitals, and all the patients signed the informed consent at the time of hospitalization.

## 3. Results

### 3.1. Demographic and Clinical Characteristics

In the mentioned period, we identified 50 cases of large B-cell lymphoma, of which 15 cases were eligible for our study, for each of the following localizations in the gastrointestinal tract: oropharyngeal region, stomach, small intestine, and large bowel.

After applying the eligibility criteria, 15 cases were identified, of which 86.67% were DLBCL, NOS, and 13.33% were HGBL.

The most frequent localization of DLBCL, NOS, in the GI tract, was in the stomach (33.33%) and the small intestine (33.33%). Also, the most frequent localizations of DLBCL, NOS, in the GI tract of both males and females, with the same percent, were in the stomach (33.33%) and the small intestine (33.33%). 

The average age of the patients was 56.73 years (SE 3.833) with extremes between 32 and 86 years. The male gender was the most affected, observed in 60% of cases. The most common comorbidities encountered were secondary anemia (93.33%) and hyperuricemia (20%). A statistically significant association was observed between the presence of secondary anemia and young patients, unlike the older ones (*p* = 0.035). As a significant risk factor for the development of the disease, only one single case presented infection with the Helicobacter pylori bacterium and DLBCL, NOS, localized in the stomach (6.67%).

In cases where the cell of origin could be identified (86.67%), a predominance of germinal center B-cell-like subtype (GCB) was noted (84.62%). The average age of patients with DLBCL, NOS, was higher (58.15 years) compared to the average age of those with HGBL (47.50 years), without a statistically significant difference (*p* = 0.305). A slight predisposition of the male sex for DLBCL, NOS, and HGBL for the female sex was observed, but without a statistically significant correlation (*p* = 0.143).

The most common variant of DLBCL was centroblastic (80%). Patients with this form have an average age older than the others (57.58 years).

The distribution at the level of the digestive tract exhibited an increased frequency of them at the level of the stomach and the small intestine (33.33% each), less often evident at the proximal level (oropharyngeal 13.33%). Depending on the lesional topography, increased survival was observed in the locations in the large intestine (94.20 weeks), unlike the others (*p* = 0.011). However, location does not represent an independent risk factor predicting mortality (*p* = 0.054).

The average LDH value was 731.07, with limits between 164 and 3956. In 66.67% of cases, a value above the normal limit was observed. A statistically significant difference was observed, consisting of the fact that the advanced age of the patients correlated with the increased LDH values (*p* = 0.040). In cases of DLBCL, NOS, a much higher average of LDH values was observed, in contrast to cases with HGBL (798.31 vs. 294), without statistical significance (*p* = 0.561). Regarding the survival of the patients according to this parameter, no major differences were observed—31.39 weeks for increased values and 33.66 for normal values.

Most of the patients (73.3%) had a low IPI score (0–2), and more than half of the patients (53.3%) had a high ECOG (2–4) prognostic score. Ann Arbor staging highlighted all cases in the high-grade category (III–IV). 

Almost all the patients with lymphoma in the small bowel had a high IPI score (3–5) compared to the other locations, and in contrast, all the patients with lymphoma located in the stomach had a low IPI score (0–2) (*p* = 0.012). 

The same aspect was found and could be transposed in the ECOG prognostic score where a high-grade ECOG (2–4) was associated with small intestine localization, but with no statistical significance (*p* = 0.073). 

No statistically significant correlation was observed between Ki67 expression analyzed with the conventional method or AI method and ECOG performance status, or Ann Arbor staging. 

In the case of patients with DLBCL, NOS, the average survival was 29.93 weeks, while in the case of those with HGBL, the survival was 40.63 weeks, without statistical significance (*p* = 0.954).

### 3.2. The Proliferation Index, Treatment, and Survival Rate 

The average proliferation index (Ki67) was 58.33% with values between 10% and 85%. However, visual assessment provides lower Ki67 values than automated digital image analysis (see Table 1).

**Figure 5:**
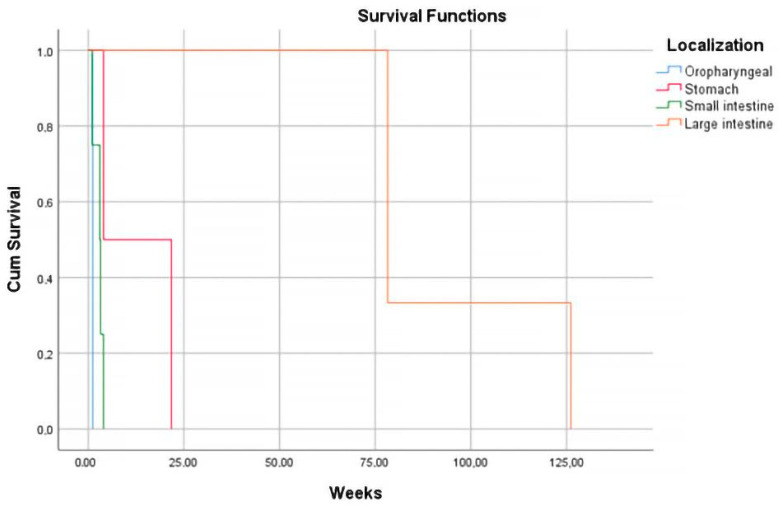
Kaplan–Meier survival graphic shows a higher survival rate for lymphomas localized in the large intestine.

After the stratification of the cases, an increased proliferation index was observed in the majority of cases (53.33%). This aspect was associated with the advanced age of the patients (*p* = 0.045). In cases of HGBL, a higher average proliferative index was observed in contrast to DLBCL (80% vs. 55%), without statistical significance (*p* = 0.114). A difference without statistical significance was observed between the proliferative index and location, and thus the lesions of the large intestine presented an increased multiplication ratio, and at the oropharyngeal level, the lowest proliferation rate was observed (*p* = 0.688) (Figure 5). However, whether it was an index classified as high or low, the average survival was similar (31.04 weeks and 33.62 weeks, respectively). 

The agreement coefficient between the conventional method and the AI method indicates an excellent level of reliability (ICC1–0.970, ICC2–0.990), data that can be consulted in Table 2.

All patients benefited from treatment, the most common scheme being CHOP (53.33%). Patients treated with this scheme had an average survival of 26.66 weeks, and those with R-CHOP had an average survival of 44.69 weeks, without statistical significance (*p* = 0.302).

At the end of the study, 10 patients died (66.67%). The average life span of the patients in the group who died was 32.07 weeks (1–126.09 weeks).

Univariate analysis of the IPI score and Ki67 evaluation in the studied group of patients revealed no statistically significant correlation. Otherwise, the multivariate analysis revealed that in the cases where the proliferation index Ki67 is high (˃70%), the IPI score represents an important risk factor predicting mortality (HR = 10.597, *p* = 0.033), and data can be consulted in Table 3. 

## 4. Discussion

Large B-cell lymphomas, with an estimated 150,000 new cases annually worldwide, represent almost 30% of all cases of non-Hodgkin lymphoma [3,17]. The updated World Health Organization (WHO) classification has refined the categorization of large B-cell lymphomas, which are a heterogeneous collection of clinicopathological entities that diffuse large B-cell lymphoma, not otherwise specified (DLBCL, NOS), is the most common [17]. In our study, DLBCL, NOS, was the most common in the group of patients studied (86.67%), in alignment with the results obtained with the data provided by WHO 2022. 

According to the studies of Bautista-Quach et al., Ghimire P. et al., and Olszewska-Szopa et al., the most frequent site for gastrointestinal NHL is the stomach (60–75% of all cases), followed by the small intestine and the ileocecal region, and two of the most prevalent diagnoses are diffuse large B-cell lymphoma (DLBCL) and marginal zone lymphoma (MALT) [18,19,20]. Our research supports this; the patients’ most frequent localization of DLBCL, NOS, in the GI tract was in the stomach (33.33%) and the small intestine (33.33%). 

According to Sehn et al., the median age at diagnosis of DLBCL is in the mid-60s, and 30% of patients are older than 75 years of age [17]. In our study, the median age of patients at diagnosis of DLBCL, NOS, was under those values (58.15 years).

In the World Health Organization (WHO) Classification of Tumors of Hematopoietic and Lymphoid Tissues, information on the cell of origin (COO), either by immunohistochemical (IHC) stainings or by gene expression profiling (GEP), is required for a definite DLBCL diagnosis [13,20,21,22,23,24]. As for the molecular subtypes, the GCB subtype has a frequency of about 60%, while the subtype ABC has a frequency of about 25–30% [17]. Our research identified the cases where the cell of origin could be identified (86.67%), with a higher predominance of the GCB subtype (84.62%).

DLBCL, NOS, occurs in men slightly more frequently than women [3]. In our case, similarly, the male gender was the most affected, observed in 60% of cases. Also, in comparison with HGBL, a slight predisposition of the male sex for DLBCL, NOS, and HGBL for the female sex was observed, but without a statistically significant correlation (*p* = 0.143).

Globally, the current treatment of patients with R-CHOP (rituximab, cyclophosphamide, doxorubicin, vincristine, prednisone) has been the standard of care in the first line for DLBCL [25,26]. Yet, 20% to 50% of DLBCL patients experience relapse or are refractory to the first line and become eligible for second-line and subsequent treatment [27,28]. Unfortunately, once a patient reaches the third line, few treatments have been available historically, and overall survival is less than 30.43 weeks [29]. Our research identified the fact that patients treated with this scheme had an average survival of 26.66 weeks, and those with R-CHOP had an average survival of 44.69 weeks, without statistical significance (*p* = 0.302).

According to several recent studies by Huang et al. and Scherer et al., LDH is an effective marker of tumor bulk in solid and hematological malignancies [30,31]. The LDH value is a contributing factor to the International Prognostic Index (IPI) and serves as a helpful marker of the severity of the illness and the effectiveness of treatment. However, recent studies have shown that in lymphoma, LDH correlates strongly with higher levels of cell-free tumor DNA and might be a surrogate of increased circulating tumor cells [31,32]. The present study demonstrates that in 66.67% of cases, a value above the normal limit (>250) and a statistically significant difference were observed, consisting of the fact that the advanced age of the patients correlated with the increased LDH values (*p* = 0.040). In our research, regarding the survival of the patients according to this parameter, no major differences were observed—31.39 weeks for increased values and 33.66 for normal values. 

According to a study by Prochazka et al., in univariable time-to-event analysis, elevated uric acid levels were associated with a worse PFS (hazard ratio (HR)) and a worse OS [33]. In our study, one of the most common comorbidities encountered was hyperuricemia (20%) but did not correlate with a worse OS. 

Nuclear protein Ki-67 is involved in controlling cell proliferation, and its expression has been commonly used as a marker to assess lymphoma proliferative activity. Its predictive significance for lymphoma is still undefined and insufficient, nevertheless [15]. According to several studies, high Ki-67 expression was linked to worse event-free survival (EFS), progression-free survival (PFS), and overall survival (OS) in DLBCL, as well as a higher International Prognostic Index (IPI) [13,34,35,36,37,38]. In our study, after the stratification of the cases, an increased proliferation index was observed in the majority of cases (53.33%), and this aspect was associated with the advanced age of the patients (*p* = 0.045). The average survival was similar (31.04 weeks and 33.62 weeks, respectively) whether the index was classified as high or low. In cases of HGBL, a higher average proliferative index was observed in contrast to DLBCL (80% vs. 55%), without statistical significance (*p* = 0.114). 

In a study by Llanos et al., high Ki-67 expression was associated with extranodal involvement [39]. In our research, a difference without statistical significance was observed between the proliferative index and location, and thus the lesions of the large intestine presented an increased multiplication ratio, and at the oropharyngeal level, the lowest proliferation rate was observed (*p* = 0.688) (Figure 5). 

According to a study by Broyde et al., in diffuse large B-cell lymphoma, a cut-off value of 70% can distinguish patients with a good and bad prognosis when combined with other prognostic factors of low IPI score and bulky disease [40]. Similarly, our study has shown that in large B-cell lymphoma cases where the proliferation index Ki67 is high (˃70%), the IPI score represents an important risk factor in predicting mortality.

However, the intra- and interobserver variability of Ki-67, which is dependent on the heterogeneity of the tumor and the area under examination, discourages it from being validated as a prognostic factor [41]. In our study, the agreement coefficient between the conventional method and the AI method indicates an excellent level of reliability (ICC1–0.970, ICC2–0.990). 

A peculiarity of this study, without statistical significance but drawing attention to the deceptive aspect of this pathology, consists of the difference between the proliferative index and localization (*p* = 0.688). The lesions identified in the large intestine showed an increased multiplication ratio, while those in the oropharynx had a lower proliferation rate. However, patient survival was higher in the case of localization in the large intestine.

According to Bankhead et al., manual subjective scoring of these data by traditional pathologist assessment is no longer sufficient to support large-scale tissue biomarker trials and cannot ensure the high-quality, reproducible, objective analysis essential for reliable clinical correlation and candidate biomarker selection [7]. In practice, QuPath’s built-in cell segmentation algorithms can detect potentially millions of cells as objects within a single WSI, in addition to measuring cell morphology and biomarker expression, and evaluate the presence, localization, and intensity of expression of essential diagnostic, prognostic, and predictive biomarkers in tissue sections. As a result of the extensive phenotypic description of each cell, the entire tissue section provides a quantitative cellular map that can be used to pick, query, and filter the image data to mine them for morphological details that would not normally be seen during the standard pathological examination. All of this can typically be achieved within minutes, without a requirement for specialist hardware [7]. 

Tissue microarrays (TMAs) are an increasingly popular and influential resource for assessing the relationship of biomarkers, including Ki67, with outcomes in large phase III clinical trials or epidemiological studies [42]. Most data in the literature are derived from visual scoring, which may be aided by the use of a grid [42]. Digital imaging may be helpful, but because all stained malignant cells are regarded as positive, irrespective of the intensity of the stain, the contribution of imaging to the removal of subjective bias is less important for Ki67 than with some markers. Also, the loss of integrity of the interior of nuclear material may make the selection of positive nuclei more difficult for some image analysis systems [42]. Hence, these observations are consistent with the results of our research.

Regarding the results obtained in this study, they are preliminary, and other factors may be involved, such as genetic factors (e.g., alterations, mutations). Further multicenter study with more DLBCL cases is desirable soon.

## 5. Conclusions

As a peculiarity of this study, the most frequent localizations of DLBCL, NOS, in the GI tract of both males and females, with the same percent, were the stomach and the small intestine, and the proliferative index of the lesions of the large intestine presented an increased multiplication ratio with a higher survival rate, while at the oropharyngeal level, it showed the lowest proliferation rate. 

In conclusion, our study has shown that in the large B-cell lymphoma cases where the proliferation index Ki67 is high (˃70%), the IPI score represents an important risk factor predicting mortality, with no statistically significant correlation between Ki67 expression analyzed with the conventional method or AI method and ECOG performance status, or Ann Arbor staging. Despite the challenging histopathologic screen for practicing pathologists since different forms of lymphomas exhibit modest heterogeneity in their histologic findings, automatic identification was found to be more objective and reproducible. The assessment of Ki67 in non-Hodgkin large B-cell lymphomas with the aid of QuPath Software can reach the goal of increasing the productivity of pathologists and can demonstrate the feasibility of integrating an automated lymphoma diagnostic screen to help in giving more accurate diagnostics of lymphoma in the pathology workflow in the future. Future studies with larger patient cohorts are needed to elucidate the diagnostic utility of digital pathology and the prognostic role of Ki67 in patient survival in patients with large B-cell NHL.

## Figures and Tables

**Table 1:** Ki67 evaluation values by using conventional method controls and QuPath software.

	Ki67 Evaluation			
No. of Case	Conventional Method (Controls) Low (≤70%)/High (>70%)		QuPath Software Evaluation	
	Pathologist 1 N (%)	Pathologist 2 N (%)	Positive Cells (%)	The Mean Value of Positive Cells
1	70%	75%	73.69%	79.870
2	70%	75%	72.96%	78.960
3	40%	45%	37.13%	58.440
4	85%	80%	88.99%	85.876
5	20%	20%	22.67%	19.978
6	75%	80%	79.97%	89.258
7	80%	75%	71.78%	75.786
8	50%	60%	62.58%	68.691
9	75%	75%	67.70%	71.637
10	50%	50%	49.67%	53.667
11	80%	85%	83.80%	86.356
12	40%	45%	42.05%	58.015
13	30%	25%	35.52%	65.037
14	65%	70%	61.29%	58.184
15	30%	30%	33.03%	64.716

**Table 2:** Inter-rater reliability between the conventional method and the QuPath software.

	ICC	95% Confidence Interval		F Test with True Value 0			
		Lower Bound	Upper Bound	Value	df1	df2	Sig
Single Measures	0.970	0.932	0.989	100.251	14	28	0.000
Average Measures	0.990	0.976	0.996	100.251	14	28	0.000

**Table 3:** Univariate and multivariate Cox regression analyses revealed the association between the IPI score and the proliferation index Ki67.

	Univariate Analysis			Multivariate Analysis		
	HR	CI95	*P*	HR	CI95	*p*
IPI score	5.406	0.945–30.932	0.058	10.597	1.211–92.717	0.033
Ki67 Low/High	1.122	0.278–4.528	0.871	2.888	0.479–17.424	0.248

## Data Availability

All data generated or analyzed during this study are included in this published article.

## Abbreviations

ABC	activated-B-cell-like
AI	artificial intelligence
COO	cell of origin
CHOP	cyclophosphamide, hydroxydaunorubicin, oncovin (vincristine), prednisone, or prednisolone
CI	confidence interval
DLBCL, NOS	diffuse large B-cell lymphoma not otherwise specified
DP	digital pathology
EFS	event-free survival
FDA	Food and Drug Administration
FL	follicular lymphoma
GCB	germinal center B-cell-like
GI	gastrointestinal
GEP	gene expression profiling
HGBL	high-grade B-cell lymphoma
HR	hazard ratio
LPD	lymphoproliferative disorders
LDH	lactate dehydrogenase
MALT	marginal zone lymphoma
NA	North America
NON-GCB	non-germinal center B-cell-like
NHL	non-Hodgkin lymphoma
OS	overall survival
PFS	progression-free survival
R-CHOP	rituximab, cyclophosphamide, hydroxydaunorubicin, oncovin (vincristine), prednisone, or prednisolone
SEEU	South-Eastern Europe
WEU	Western Europe
WHO	World Health Organization
WSI	whole slide imaging
